# SERS‐based immunomagnetic bead for rapid detection of H5N1 influenza virus

**DOI:** 10.1111/irv.13114

**Published:** 2023-03-09

**Authors:** Xiwen Wang, Song Li, Han Qu, Liangyu Hao, Ting Shao, Kai Wang, Zhiping Xia, Zhiping Li, Qianxue Li

**Affiliations:** ^1^ Changchun Veterinary Research Institute Chinese Academy of Agricultural Sciences Changchun China; ^2^ Administration for Drug and Instrument Supervision and Inspection of PLAJLSF Beijing China; ^3^ Jilin Province Animal Husbandry and Veterinary Academy of Sciences Changchun Jilin China; ^4^ The People's Hospital of Changchun Changchun China

**Keywords:** H5N1 influenza virus, label‐free, SERS

## Abstract

The surface‐enhanced Raman scattering (SERS) has recently drawn attention in the detection of respiratory viruses, but there have been few reports of the direct detection of viruses. In this study, a sandwich immunomagnetic bead SERS was established for the rapid diagnosis of the H5N1 influenza virus. The detection limit was estimated to be 5.0 × 10^−6^ TCID_50_/ml. The method showed excellent specificity with no cross‐reaction with H1N1, H5N6 or H9N2. The H5N1 influenza virus detection accuracy of the SERS method was 100% in chicken embryos. The results hold great promise for the utilization of SERS as an innovative approach in the diagnosis of influenza virus.

## 1 INTRODUCTION

**FIGURE 1:**
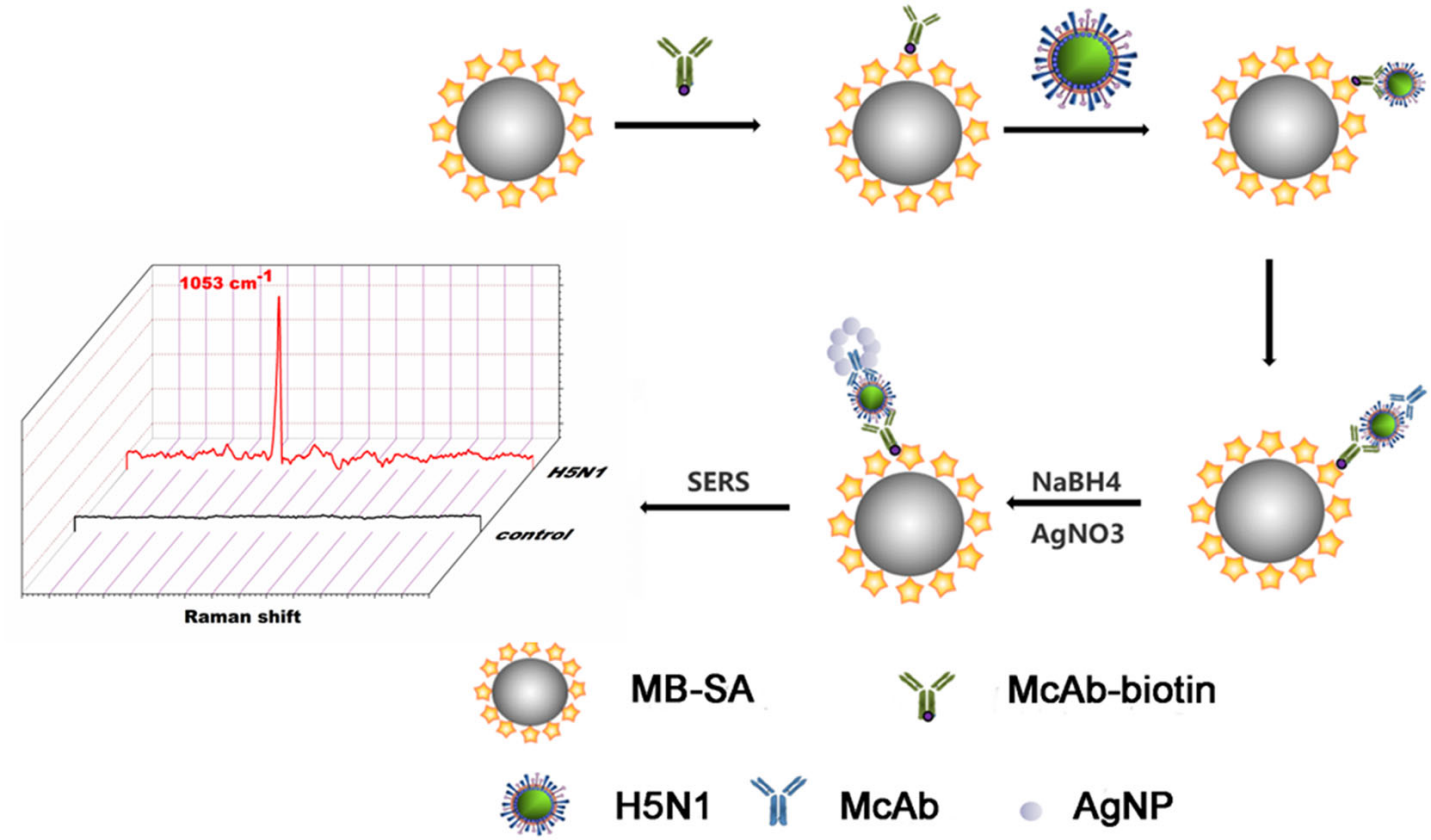
Schematic of the IMBSIs@Ag‐SERS method for H5N1 influenza virus detection.

The influenza A virus of the H5N1 subtype is a highly pathogenic avian influenza (HPAI) that leads to severe acute respiratory infection in humans, with a fatality rate of more than 50%.^1^ An efficient analytical method capable of the reliable detection and identification of viruses is necessary for the adequate management of epidemic. Accurate analytical techniques or rapid detection assays are used for virus identification or quantification. The commonly used methods to detect or quantify viruses can be subdivided into virus culture, serological test, nucleic acid‐based detection methods and biosensors‐based point‐of‐care testing system.^2^ The virus culture and reverse transcription PCR (RT‐PCR) had been the gold standard lab methods for the H5N1 influenza virus detection.^3^ Real‐time PCR presents high specificity, sensitivity, stability and experimental reliability and is currently used to detect influenza viruses and other pathogens. However, complex processes and long sample preparation times are required of these detection methods. Surface‐enhanced Raman scattering (SERS) as a molecular fingerprint spectrum has been applied to a variety of influenza A virus subtypes, such as the H1N1, H7N9, H3N2, H5N1 and the novel coronavirus SARS‐CoV‐2.^4^ In the present study, we established a highly sensitive and label‐free SERS detection method for tracking the Raman signal of the H5N1 influenza virus (Figure 1). The H5N1 influenza virus was shown to bond with a biotinylated primary antibody on the magnetic beads and later was combined with a secondary antibody to form an immunomagnetic beads sandwich immunocomplexes (IMBSIs). The strong SERS signal of the H5N1 influenza virus can be sensitively detected through the in‐situ reduction of nano‐silver serving as a SERS substrate.

## 2 MATERIALS AND METHODS

### 2.1 Virus and Raman instrument

Avian influenza viruses (A/chicken/Jilin/9/2004/H5N1) were obtained from our laboratory stock. The H5N1 influenza virus titer was 5.0 × 10^6^ TCID_50_/ml (50% tissue culture infective dose), as determined by the monitored cytopathic effects (CPEs) of the infected cells according to standard procedures.^5^ The influenza viruses strains H1N1 (A/PR/8/34), H5N6 (A/chicken/Hubei/165/2016) and H9N2 (unnamed) were gifted by Dr. Guo (Changchun Veterinary Research Institute, Chinese Academy of Agricultural Sciences, China). All work with the live avian influenza virus was conducted in the Biosafety Level 3 (BSL‐3) Laboratory, while the virus samples used in the current SERS study were taken from the BSL‐3 Laboratory after verification of complete inactivation. All work with the inactivated avian influenza virus was conducted in the BSL‐2 Laboratory. The Raman spectra were collected and measured using a LabRam HR evolution laser confocal Raman microprobe (HORIBA, Tokyo, Japan). The samples were observed from the Raman microscope and focused under 50 × objective. The SERS spectra were recorded using a He–Ne 532 nm laser with 14 mW. The SERS spectra were recorded with exposures of 2 s in 10 repetitions, the accumulation was two times, the width of confocal slit was 100 μm and the detecting spectra range was 500–2000 cm^−1^.

### 2.2 Preparation of immunomagnetic beads

The BeaverBeads Streptavidin magnetic beads (MB‐SA) (BeaverBeadsTM Streptavidin, BeaverBio company, Suzhou, China) were resuspended in the original vial by rotation for 20 s. MB‐SA (100 μl) were washed with phosphate buffer saline (PBS, containing 0.05% Tween‐20) three times by a magnetic separation process. Then, 1 μg/ml, 0.5 μg/ml, 0.33 μg/ml, 0.25 μg/ml and 0.1 μg/ml of biotinylated rabbit anti‐H5N1 antibody (Sino Biological Inc., Beijing, China) were added to the MB‐SA and incubated for 1 h at room temperature with gentle rotation in the tube. The antibody‐conjugated immunomagnetic beads (IMBs) were washed three times before magnetic separation experiments. After washing three times with PBS buffer, the reaction mixture was treated with bovine serum albumin (BSA, 2%) at 37°C for 1 h.

### 2.3 Preparation of sandwich immunomagnetic beads

The H5N1 influenza virus underwent 10‐fold serial dilutions in PBS and was subsequently incubated with IMBs for 30 min at 4 °C with gentle shaking. Different concentrations (dilutions) of the virus were used for detecting sensitivity. The immunocomplexes were then washed with PBS buffer solution containing 0.05% Tween‐20 three times to remove unbound viruses. Then, 0.5 μg/ml of detection anti‐H5N1 hemagglutinin monoclonal antibody (Sino Biological Inc., Beijing, China) was added for 30 min at room temperature. Finally, the conjugated IMBs sandwich immunocomplexes (IMBSIs) were pipetted for washing in PBS and subsequent magnetic separation.

### 2.4 In situ reduction of nano‐silver and TEM detection

The in situ reduction of nano‐silver was used for synthesis according to our previously study.^6^ Briefly, 50 μl of IMBSIs was centrifuged at 3000 rpm for 5 min, and the supernatant was discarded. Then, 50 μl (1.72 mg/ml) of AgNO_3_ was mixed with 50 μl of IMBSIs for 5 min. Next, 50 μl of a 0.378 mg/ml NaBH_4_ solution was rapidly added, vortexed and centrifuged, and the supernatant was discarded. The obtained samples were stored at 4 °C and were stable for months without light. Microstructures of the magnetic beads and IMBSIs in the in‐situ reduction of nano‐silver (IMBSIs@Ag) were investigated by transmission electron microscopy (TEM).

### 2.5 SERS measurements of H5N1 influenza virus

The SERS measurements were performed in different samples, including H5N1 influenza virus in IMBSIs@Ag‐SERS, H5N1 influenza virus in IMBSIs, IMBSIs@Ag‐SERS, H5N1 influenza virus in IMBs@Ag‐SERS, hemagglutinin protein in IMBs@Ag‐SERS and IMBs@Ag‐SERS. The Raman spectrum of the hemagglutinin protein in power and H5N1 influenza virus can be detected without SERS in same condition. The SERS method was used to compare the binding efficiency of the different concentrations of the primary antibodies in capturing the H5N1 influenza virus. The specificity of the detection method was obtained by comparing influenza viruses H1N1, H5N6 and H9N2 (replacing H5N1 in IMBSIs@Ag for SERS). The H1N1, H5N6, H9N2 or H5N1 influenza virus titer was 5.0 × 10^6^ TCID_50_/ml, as determined by the monitored cytopathic effects of the infected cells. The repeatability of the method was observed in three independent H5N1 influenza virus samples. The SERS spectra were repeated three times for each different sample. The limit of detection (LOD) of SERS was calculated for different concentrations of H5N1 influenza virus.

### 2.6 Identification of H5N1 influenza virus from chicken embryos

To validate the performance and specificity of the IMBSIs@Ag‐SERS method for rapid detection of the H5N1 influenza virus in infected and uninfected allantois fluid. The allantois fluid of 12 randomized samples from 200 infected and 200 uninfected SPF chicken embryos was confirmed by the HA (hemagglutination) test and the IMBSIs@Ag‐SERS method. An HA test was performed in 96‐wells coagulation with 1% chicken red blood cells.

## 3 RESULTS

### 3.1 Working principle of the IMBSIs@Ag‐SERS method

**FIGURE 2:**
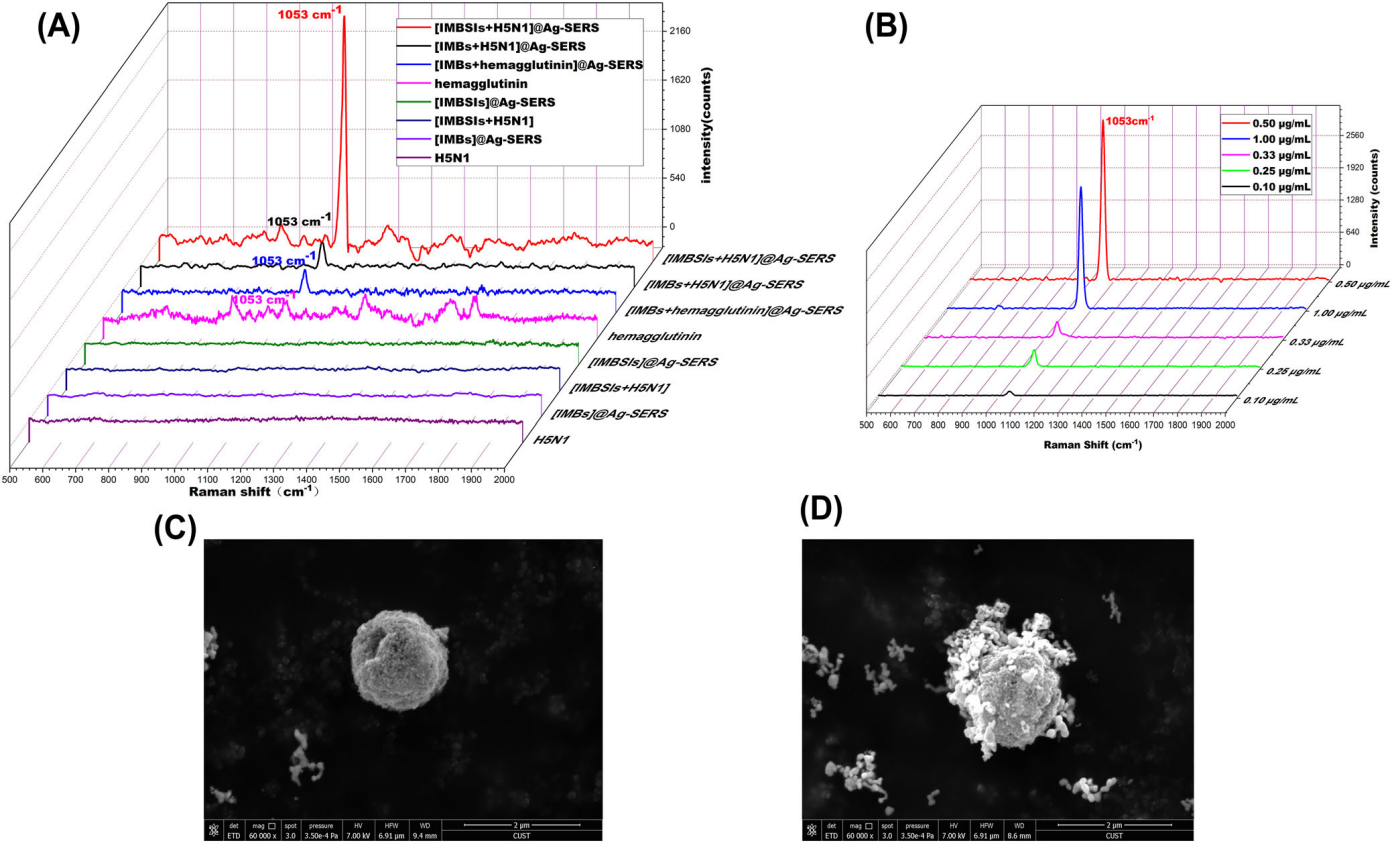
Detection of H5N1 influenza virus by IMBSIs@Ag‐SERS method. (A) Effect of SERS enhancement in situ reduction of nano‐silver. (B) SERS of H5N1 influenza virus captured by the primary antibodies in different concentrations. (C) TEM of naked magnetic beads with streptavidin in situ reduction of nano‐silver. (D) TEM of immunomagnetic beads sandwich immunocomplexes (IMBSIs) in situ reduction of nano‐silver.

In this study, we used IMBs composed of antibodies as molecular recognition elements to capture the H5N1 influenza virus, and then the second antibody was added to the IMBs to form sandwich immunocomplexes. This structure is referred to as IMBSIs for the in‐situ reduction for SERS of silver nanoparticles. The Raman spectra of IMBSIs@Ag‐SERS+H5N1 showed a prominent peak at 1053 cm^−1^ compared to the unenhanced IMBSIs+H5N1 and the enhanced IMBSIs@Ag‐SERS or IMBs@Ag‐SERS (Figure 2A). It showed excellent capturing efficiency at 0.5 μg/ml of the primary antibody concentration (Figure 2B). It can be clearly observed from the TEM image that nano‐silver was synthesized around the surface of IMBSIs (Figure 2C, D), which resulted in the specific amplified signal. The results display that the Raman peak could be used for quantitative determination of viruses using the example of influenza A virus.

### 3.2 Specificity, uniformity and repeatability of SERS assay

**FIGURE 3:**
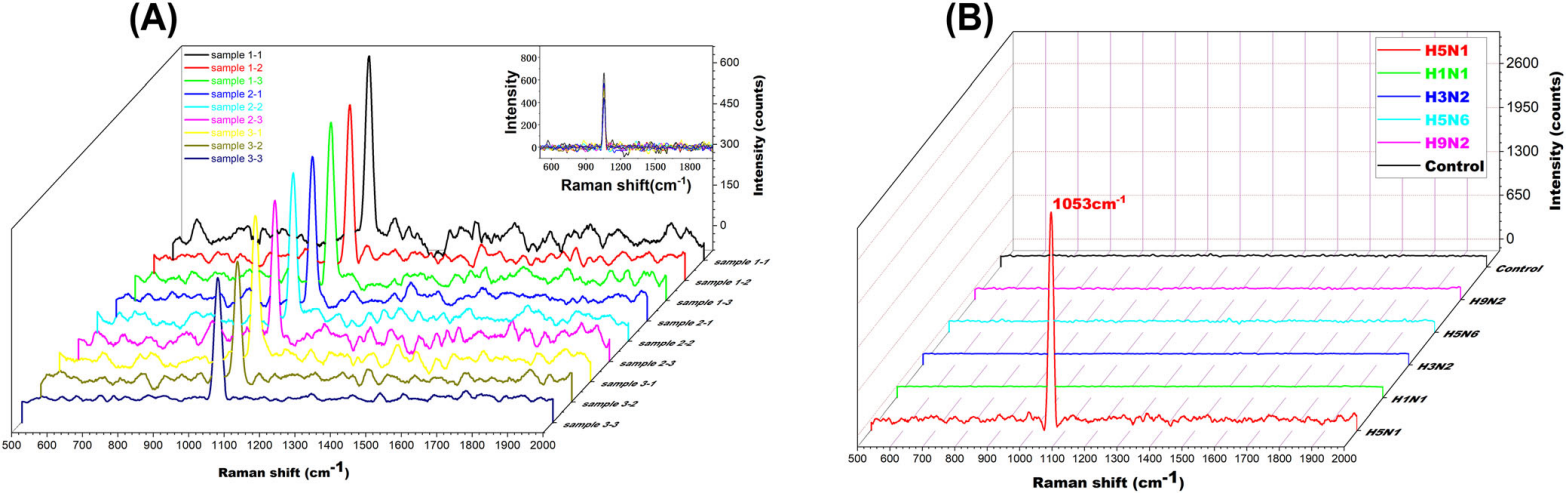
Excellent repeatability and specificity of SERS detection in H5N1 influenza virus. (A) The IMBSIs@Ag SERS showed a consistent Raman intensity at 1053 cm^−1^ for nine times. (B) The IMBSIs@Ag SERS no characteristic peak at 1053 cm^−1^ replacing H5N1 with H1N1, H5N6 or H9N2.

**FIGURE 4:**
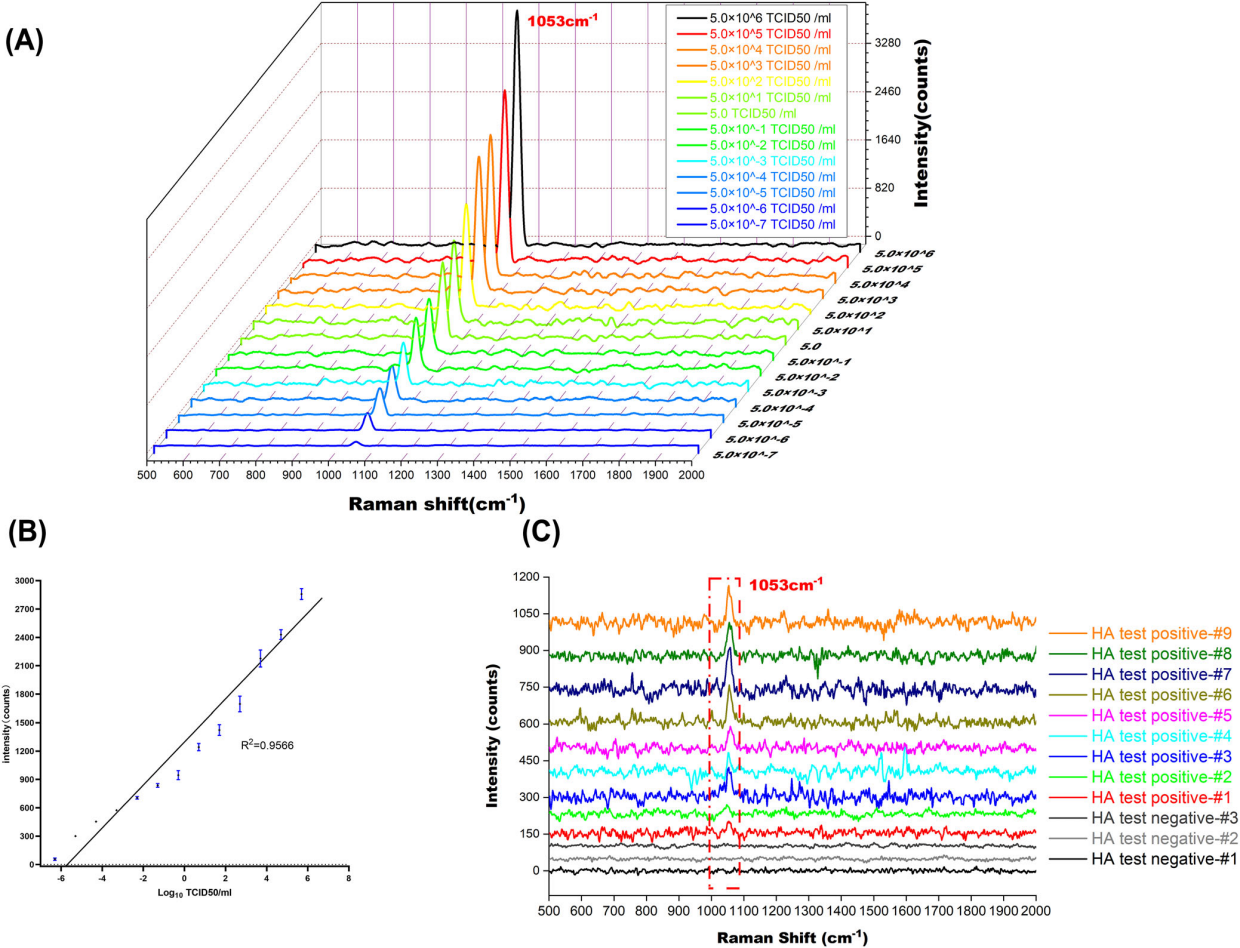
The susceptibility of IMBSIs@Ag for detection of the H5N1 influenza virus. (A) IMBSIs@Ag Raman intensity of the H5N1 influenza virus was 10‐fold diluted from 5.0 × 10^6^ to 5.0 × 10^−7^ TCID_50_/ml. (B) Linear relationship between Raman intensity and H5N1 influenza virus concentration. (C) IMBSIs@Ag SERS identification of H5N1 influenza virus from chicken embryos.

The result showed a consistent Raman intensity at 1053 cm^−1^ in three independent H5N1 influenza virus samples, which were performed again in triplicate (Figure 3A). Influenza viruses H1N1, H5N6 or H9N2 were used instead of H5N1 in IMBSIs@Ag for the specificity test. Influenza viruses H5N6 and H5N1 have similar HA protein, while influenza virus H1N1 and H5N1 have the similar NA protein, which make them suitable for specific evaluation. The H5N1 influenza virus detection result of Raman spectrum performance showed excellent repeatability and specificity (Figure 3B). The H5N1 influenza virus was 10‐fold diluted from 5.0 × 10^6^ to 5.0 × 10^−7^ TCID_50_ /ml. As shown in Figure 4A, the limit of detection (LOD) is 5.0 × 10^−6^ TCID_50_/ml in Raman spectra. The Raman peak height reduced when the H5N1 influenza virus concentration decreased, and the calibration showed a linear dependence between the virus concentration and the Raman peak height at 1053 cm^−1^ (R^2^ = 0.9566) (Figure 4B).

### 3.3 Identification of H5N1 influenza virus from chicken embryos

To demonstrate the applicability of the SERS in IMBSIs@Ag in allantois fluid with the H5N1 influenza virus infected and uninfected. The results were consistent with Raman spectra and HA tests. Twelve samples were randomly selected from 200 infected and 200 uninfected chicken embryos. Nine of these samples tested positive, and three samples tested negative for the H5N1 influenza virus in the HA test (data aren't shown). The Raman spectra results showed a peak at 1053 cm^−1^ from nine samples but no peak for three of the samples from twelve random samples (Figure 4C). The detection accuracy of Raman spectroscopy using the IMBSIs sandwich method was 100%.

## 4 DISCUSSION

A wide variety of SERS‐based schemes were developed to detect viruses due to the different types of signal‐enhancing substrates.^7^ Most of them consumed antisense oligonucleotides to capture the genome of the virus.^8^ Biorecognition molecules (antibodies, complimentary nucleic acids and aptamers) can be functionalized onto the surface of the nanoparticles to serve as Raman reporters.^9^ We have developed a label‐free SERS detection of the H5N1 influenza virus. Depending on the characteristics of antibody‐virus‐antibody interactions, the H5N1 influenza virus was captured with primary antibody in immunomagnetic beads. The secondary antibody was added, followed by the in situ reduction of silver nanoparticles. Interestingly, the IMBs captured H5N1 and showed a narrow peak at 1053 cm^−1^ without the presence of the second antibody, but had a lower sensitivity than the IMBSIs captured H5N1. There was a gap between the sample and nano‐silver, which enhanced the SERS effect. The gap width was the distance of the second antibody that perhaps produced the electromagnetic enhancement in the Raman signal. Influenza hemagglutinin (HA) protein is a glycoprotein found on the surface of influenza viruses. Therefore, we attempted to replace influenza virus H5N1 with H5N1 HA protein in IMBs. The data show that there is still an obvious Raman peak at 1053 cm^−1^. Some Raman peaks at different Raman shift can be observed in HA protein without SERS (Figure 2A). The IMBs sandwich immunocomplexes enhanced the peak intensity of H5N1 at 1053 cm^−1^ compared to the rest of the spectrum.

In another study, Moon et al. utilized immunoreaction to capture the influenza A/CA/07/2009 (pH1N1) virus on a sensing platform aided by the SERS antibody tag and the limit of detection (LOD) is 4 × 10^3^ TCID_50_ /ml.^10^ Previous studies have reported a portable platform was demonstrated that the detection could be achieved with as little as 10^2^ EID_50_/mL differentiate between two avian influenza virus subtypes (H5N2 and H7N2) and reovirus, using principal components analysis (PCA).^11^ Regarding SERS studies, Wang et al. reported the quantification of the H5N1 antigen on a digital microfluidic (DMF) platform, claiming good performance when evaluating the H5N1 antigen concentration in the serum with a LOD of 74 pg/ml. In this platform, SERS tags are labelled with Raman reporters and 4‐mercaptobenzoic acid (4‐MBA). By labelling the beads‐antibody–antigen immunocomplex with a 4‐MBA SERS tag, H5N1 influenza virus can be detected through the SERS signal.^12^ However, the quantification is based on tracking the Raman signal from the reporter molecule 4‐MBA instead of the Raman signal from H5N1 influenza virus. Our study has higher sensitivity and a much lower detection limit (LOD = 5.0 × 10^−6^ TCID_50_/ml). The result also confirms our conjecture that AgNP grows on the secondary antibodies but not on the primary antibodies, leading to the outermost components of organisms being enhanced. Raman reporters can be detected rapidly and conveniently, but they are easy to lose or be quenched. The ultrasensitive sandwich method directly detected the sample, but not the Raman reporters. The Raman peaks were generated from enhanced sandwich immunocomplexes but not from the immunomagnetic beads.

The SERS method has been widely used in biological detection for its fast, accurate and fingerprint characteristics. Some researchers have shown the detection of bacteria by SERS spectra directly.^13^ Viruses differ from bacteria in that they depend on host cells for survival, replication and propagation, and it is difficult to acquire pure viruses with no impurities (contents and medium of host cells) like bacteria. Some researchers have quantitatively assayed these samples by analysing the peak height or peak area ratios, but the major quantitative analysis is based on the enhanced signals in the material (noble metals and compounds) or method (self‐assembled monolayer layers and SERS tags^14^, ^15^, ^16^). Although the result showed a linear relationship between the peak height and the virus concentration, the virus was still not able to be quantified accurately. The quantitative detection of virus was exploratory in the sensitive detection of the H5N1 influenza virus in this study. It was essential for improving the quantitative ability and widespread application in the future.^17^ This label‐free sandwich method demonstrates a real applicability in using SERS for real clinical samples. The method with high enhanced ability, stability, specificity and susceptibility will be critical for the application of SERS in detection of viruses. In the future, this in‐situ Label‐Free SERS method holds great potential for applications in portable and rapid Raman detections of a variety of infectious diseases.

## AUTHOR CONTRIBUTIONS

**Xiwen Wang:** Conceptualization; data curation; formal analysis; investigation; methodology; writing—original draft; writing—review and editing. **Zhiping Li:** Project administration. **Song Li:** Data curation; formal analysis; validation. **Han Qu:** Validation. **Liangyu Hao:** Data curation; validation. **Ting Shao:** Conceptualization; data curation. **Kai Wang:** Conceptualization; funding acquisition. **Zhiping Xia:** Conceptualization; funding acquisition. **Qianxue Li:** Funding acquisition; project administration.

## CONFLICT OF INTEREST STATEMENT

There are no conflicts to declare.

### PEER REVIEW

The peer review history for this article is available at https://publons.com/publon/10.1111/irv.13114.

## Data Availability

The data that support the findings of this study are available from the corresponding author upon reasonable request.
